# Frequency-specific alternations in the amplitude of low-frequency fluctuations in chronic tinnitus

**DOI:** 10.3389/fncir.2015.00067

**Published:** 2015-10-29

**Authors:** Yu-Chen Chen, Wenqing Xia, Bin Luo, Vijaya P. K. Muthaiah, Zhenyu Xiong, Jian Zhang, Jian Wang, Richard Salvi, Gao-Jun Teng

**Affiliations:** ^1^Jiangsu Key Laboratory of Molecular and Functional Imaging, Department of Radiology, Zhongda Hospital, Medical School, Southeast UniversityNanjing, China; ^2^Center for Hearing and Deafness, State University of New York at Buffalo, BuffaloNY, USA; ^3^Medical School, Southeast UniversityNanjing, China; ^4^Toshiba Stroke and Vascular Research Center, State University of New York at Buffalo, BuffaloNY, USA; ^5^Department of Physiology, Southeast UniversityNanjing, China; ^6^School of Human Communication Disorders, Dalhousie University, HalifaxNS, Canada

**Keywords:** chronic tinnitus, Frequency, ALFF, fALFF, fMRI

## Abstract

Tinnitus, a phantom ringing, buzzing, or hissing sensation with potentially debilitating consequences, is thought to arise from aberrant spontaneous neural activity at one or more sites within the central nervous system; however, the location and specific features of these oscillations are poorly understood with respect to specific tinnitus features. Recent resting-state functional magnetic resonance imaging (fMRI) studies suggest that aberrant fluctuations in spontaneous low-frequency oscillations (LFO) of the blood oxygen level-dependent (BOLD) signal may be an important factor in chronic tinnitus; however, the role that frequency-specific components of LFO play in subjective tinnitus remains unclear. A total of 39 chronic tinnitus patients and 41 well-matched healthy controls participated in the resting-state fMRI scans. The LFO amplitudes were investigated using the amplitude of low-frequency fluctuation (ALFF) and fractional ALFF (fALFF) in two different frequency bands (slow-4: 0.027–0.073 Hz and slow-5: 0.01–0.027 Hz). We observed significant differences between tinnitus patients and normal controls in ALFF/fALFF in the two bands (slow-4 and slow-5) in several brain regions including the superior frontal gyrus (SFG), inferior frontal gyrus, middle temporal gyrus, angular gyrus, supramarginal gyrus, and middle occipital gyrus. Across the entire subject pool, significant differences in ALFF/fALFF between the two bands were found in the midbrain, basal ganglia, hippocampus and cerebellum (Slow 4 > Slow 5), and in the middle frontal gyrus, supramarginal gyrus, posterior cingulate cortex, and precuneus (Slow 5 > Slow 4). We also observed significant interaction between frequency bands and patient groups in the orbitofrontal gyrus. Furthermore, tinnitus distress was positively correlated with the magnitude of ALFF in right SFG and the magnitude of fALFF slow-4 band in left SFG, whereas tinnitus duration was positively correlated with the magnitude of ALFF in right SFG and the magnitude of fALFF slow-5 band in left SFG. Resting-state fMRI provides an unbiased method for identifying aberrant spontaneous LFO occurring throughout the central nervous system. Chronic tinnitus patients have widespread abnormalities in ALFF and fALFF slow-4 and slow-5 band which are correlated with tinnitus distress and duration. These results provide new insights on the neuropathophysiology of chronic tinnitus; therapies capable of reversing these aberrant patterns may reduce tinnitus distress.

## Introduction

Chronic subjective tinnitus is a common hearing disorder often described as a buzzing, sizzling or ringing sensation that occurs in the absence of an objective sound. Among adults roughly 12% experience tinnitus, but the prevalence rises to 40–50% among military combat personnel exposed to intense noise (McFadden, 1982; Meikle, 1997; Cave et al., 2007; Hébert et al., 2013). In severe cases, chronic tinnitus can disrupt sleep and cause emotional distress and concentration difficulties (Leske, 1981; Lockwood et al., 2002). Since the phantom sound is often perceived in the impaired ear, tinnitus was originally believed to originate in the cochlea from aberrant hyperactivity in the auditory nerve. However, since surgical section of the auditory nerve fails to eliminate or reduce tinnitus (Dandy, 1941; Baguley et al., 1992; Jackler and Whinney, 2001), the hunt for the neural generators of tinnitus has shifted to the central nervous system. On the basis of previous electrophysiological and neuroimaging studies (Lockwood et al., 1998; Kaltenbach et al., 2005), tinnitus is now believed to be generated by aberrant neural activity in the central nervous system through such mechanisms as spontaneous hyperactivity, increased burst firing, heightened neural synchrony, aberrant tonotopic maps, abnormal coupling in distributed neural networks involving both auditory and non-auditory structures and aberrant gating of conscious perception (Henry et al., 2014). While there is support for each of these, the exact mechanisms responsible for the multifaceted dimensions of tinnitus (e.g., loudness, spectral profile, tinnitus severity, and distress) remain unclear.

Since the first human electroencephalographic (EEG) recordings in the 1920s, there has been a growing interest in neural oscillatory activity which occurs over a broad frequency range (0.05–500 Hz; Gomez et al., 2004; Kuo and Yang, 2009). The oscillations occurring in neural networks extending over large area of the brain are thought to play critical roles in perception, attention, memory and cognition and disruptions in these oscillatory patterns have been linked to neurological disorders such as epilepsy, autism, and tinnitus (Buzsáki and Draguhn, 2004). More recently, spontaneous low frequency oscillations (LFO) in the 0.01–0.1 Hz range were discovered in blood oxygen level-dependent (BOLD) signals measured during resting-state functional magnetic resonance imaging (fMRI; Biswal et al., 1995). These LFO are considered physiologically meaningful, are correlated with certain components of low frequency EEG (Penttonen and Buzsáki, 2003; Mantini et al., 2007) and are thought to contribute to long-range functional connectivity (Penttonen and Buzsáki, 2003). Resting-state fMRI has proved to be a useful noninvasive technique to assess normal and pathological brain function (Fox and Raichle, 2007; Zhang and Raichle, 2010; Schmidt et al., 2013).

Most studies to date have demonstrated disrupted functional connectivity in tinnitus using resting-state fMRI (Husain and Schmidt, 2014). Functional connectivity describes the temporal synchrony or interregional cooperation between two or more spatially separate regions. Abnormal resting-state functional networks have been found in tinnitus, such as the auditory network (Burton et al., 2012; Kim et al., 2012; Maudoux et al., 2012a; Schmidt et al., 2013), default mode network (DMN; Schmidt et al., 2013; Chen et al., 2015a,b), dorsal attention network (DAN; Burton et al., 2012; Schmidt et al., 2013), ventral attention network (VAN; Burton et al., 2012). Nonetheless, these findings are based on investigations of LFO from the perspective of temporal synchrony or interregional cooperation, but fail to consider the amplitude of alternations in local or regional neuronal activity. To identify local brain areas with abnormal activity, our group used the amplitude of low-frequency fluctuations (ALFF; Biswal et al., 1995; Zang et al., 2007) to identify specific brain regions with hyper- and hypoactive BOLD signal in patients with chronic tinnitus. Importantly, our patients had normal hearing thresholds up to the extended high frequencies and no evidence of hyperacusis (Chen et al., 2014). The ALFF values (0.01–0.08 Hz) in tinnitus patients were significantly increased in several brain regions including the middle temporal gyrus (MTG), superior frontal gyrus (SFG), and angular gyrus (AG), but were decreased in the visual cortex and thalamus. Further correlation of ALFF with tinnitus features indicated that abnormal ALFF patterns in tinnitus were linked to the duration and severity of tinnitus.

Most resting-state fMRI studies have examined tinnitus-related neural activity in a broad, low frequency band between 0.01 and 0.1 Hz (Biswal et al., 1995). Some studies, however, have found that aberrant patterns of intrinsic brain activity are restricted to specific frequency bands; these sub-bands may be generated by distinct oscillators with unique properties and physiological functions some of which are related to the alpha band in EEG (Penttonen and Buzsáki, 2003; Buzsáki and Draguhn, 2004). By decomposing resting-state fMRI LFO into four distinct frequency bands [Slow-5 (0.01–0.027 Hz), Slow-4 (0.027–0.073 Hz), Slow-3 (0.073–0.198 Hz), and Slow-2 (0.198–0.25 Hz)], Zuo et al. (2010) showed that gray matter (GM)-associated LFO amplitudes primarily occurred in the slow-4 and slow-5 frequency bands. Frequency-dependent changes in the ALFF/fALFF have been found in various neuropsychiatric diseases such as amnestic mild cognitive impairment (Han et al., 2011), schizophrenia (Yu et al., 2014), Parkinson’s disease (Hou et al., 2014), and epilepsy (Wang et al., 2014). It has been confirmed that the slow-4 band has greater test–retest reliability for LFO amplitude measures and more reliable BOLD fluctuation amplitude voxels than the slow-5 band (Zuo et al., 2010), and the functional brain networks derived in the slow-4 are more reliable than those in slow-5 (Liang et al., 2012). Moreover, the slow-5 showing higher power localizes more within DMN regions such as the middle frontal gyrus (MFG) and precuneus, while the slow-4 exhibiting less power is more robust in midbrain and basal ganglia (Zuo et al., 2010; Baria et al., 2011; Han et al., 2011), indicating that individual frequency bands might be linked to specific properties (Buzsáki and Draguhn, 2004). Therefore, these researches suggest that the inherent brain functional activity is sensitive to specific frequency bands. Although abnormal LFO measures were observed in chronic tinnitus patients, an important and potentially interesting question is whether the abnormalities in the LFO signal occur within all frequency bands or only certain bands, i.e., frequency specific alternations.

To address this issue, we compared the changes of LFO amplitudes in chronic tinnitus patients using two resting-state fMRI metrics: ALFF and fractional ALFF (fALFF). ALFF measures the total power within a broad frequency range (typically 0.01–0.10 Hz; Zang et al., 2007) while fALFF measures the power within specific frequency bands divided by the total power in the entire frequency range, i.e., frequency specific, normalized amplitudes that reflect the relative contribution of each band to the total power (Zou et al., 2008). We hypothesized that (1) aberrant LFO amplitudes in tinnitus may depend on the specific frequency bands, especially the slow-4 and slow-5 bands that were mainly associated with neuronal oscillations, and that (2) these frequency specific abnormalities would be correlated with unique tinnitus characteristics such as tinnitus duration or distress.

## Materials and Methods

### Subjects

All subjects provided written informed consent before their participation in the study protocol, which was approved by the Research Ethics Committee of the Affiliated Zhongda Hospital of Southeast University.

This study was conducted from September 2011 to September 2013. A total of 82 subjects including 41 chronic tinnitus patients and 41 healthy controls were recruited through community health screenings and newspaper advertisements. The tinnitus patients and healthy subjects were group-matched in terms of age, sex, and education. Two tinnitus patients were subsequently excluded because of they exceeded the limits of head motion during MR scanning. Sixteen patients reported a predominantly left-sided tinnitus, 13 a predominantly right-sided tinnitus, and 10 patients described their tinnitus as bilateral or originating within the head. All subjects were right handed and completed at least 8 years of education. The patients ranged from 20 and 70 years of age (41.5 ± 14.6 years), with tinnitus duration of 6–120 months (36.9 ± 36.4 months). The severity of tinnitus and related distress were assessed by the Iowa version of the Tinnitus Handicap Questionnaires (THQ; Kuk et al., 1990). Hearing thresholds were determined by puretone audiometry (PTA) examination. All participants had normal hearing (thresholds <25 dB HL) in the 10 frequencies from 250 Hz to 16 kHz. There were no significant differences in auditory thresholds between the tinnitus and control groups. None of the participants had accompanied symptoms such as depression and anxiety according to the Self-Rating Depression Scale (SDS) and Self-Rating Anxiety Scale (SAS; overall scores <50, respectively; Zung, 1971, 1986). Participants were excluded from the present study if they suffered from pulsatile tinnitus, hyperacusis or Meniere’s diseases, or if they had a past history of severe smoking, alcoholism, brain injury, stroke, Alzheimer’s disease, Parkinson’s disease, epilepsy, major depression, or other neurological or psychiatric disorders that could affect cognitive function, major medical illness (e.g., anemia, thyroid dysfunction and cancer), MRI contraindications, or severe visual loss. The characteristics of the chronic tinnitus patients and healthy subjects are summarized in **Table 1**.

**Table 1:** Characteristics of tinnitus patients and healthy controls.

	Tinnitus patients (*n* = 39)	Healthy controls (*n* = 41)	*p*-value
Age (year)	41.5 ± 14.6	46.0 ± 12.2	0.137
Gender (male: female)	24:15	21: 20	0.352
Education levels (years)	10.7 ± 1.9	11.1 ± 1.5	0.545
Tinnitus duration (months)	36.9 ± 36.4	-	-
THQ score	43.5 ± 21.3	-	-

*Data are represented as mean ± SD. THQ, tinnitus handicap questionnaire.*

### MRI Acquisition

Magnetic resonance imaging data were acquired at the Radiology Department of Zhongda Hospital using a 3.0 T MRI scanner (Siemens MAGENETOM Trio, Erlangen, Germany). Head motion and scanner noise were reduced using foam padding and earplugs. The earplugs (Hearos Ultimate Softness Series, USA) were used to attenuate scanner noise by approximately 32 dB. Subjects were instructed to lie quietly with their eyes closed without falling asleep, not think of anything in particular, and avoid any head motion during the scan. Functional images were obtained axially using a gradient echo-planar imaging sequence as follows: repetition time (TR) = 2000 ms; echo time (TE) = 25 ms; slices = 36; thickness = 4 mm; gap = 0 mm; field of view (FOV) = 240 mm × 240 mm; acquisition matrix = 64 × 64; and flip angle (FA) = 90°. The resting-state fMRI scan took 8 min and 6 sec. Structural images were acquired with a T1-weighted 3D spoiled gradient-echo sequence as follows: TR = 1900 ms; TE = 2.48 ms; slices = 176; thickness = 1 mm; gap = 0 mm; FA = 90°; acquisition matrix = 256 × 256; FOV = 250 mm × 250 mm. The structural sequence took 4 min and 18 sec.

### Functional Data Preprocessing

Functional data analyses were conducted using Data Processing Assistant for Resting-State fMRI (DPARSF) programs (Chao-Gan and Yu-Feng, 2010), based on statistical parametric mapping (SPM8^1^) and resting-state fMRI data analysis toolkits (REST^2^). A total of 240 volumes were scanned, and the first 10 volumes were discarded to allow for signal equilibrium of the initial magnetic resonance signals and adaptation of the subjects to the circumstances. The remaining 230 consecutive volumes were used for data analysis. Subsequently, the following procedures were conducted in order: slice-timing adjustment, realignment for head-motion correction, spatial normalization to the Montreal Neurological Institute (MNI) template (resampling voxel size = 3 mm × 3 mm × 3 mm) and smoothing with an isotropic Gaussian kernel (FWHM = 6 mm), detrending and filtering (0.01–0.08 Hz). Any subjects with a head motion >2.0 mm translation or a 2.0° rotation in any direction were excluded.

### ALFF and fALFF Calculation

Amplitude of low-frequency fluctuation/fALFF was calculated using REST software. ALFF values were calculated through procedures described previously (Zang et al., 2007). Briefly, time courses were first converted to the frequency domain using a Fast Fourier Transform. The square root of the power spectrum was computed and then averaged across a predefined frequency interval. This averaged square root was termed ALFF at a given voxel, and was further divided by the individual global mean ALFF value to reduce the global effects of variability across participants. fALFF is the fraction of ALFF in a given frequency band divided by the ALFF over the entire frequency range (0–0.25Hz) detectable in the signal (Zou et al., 2008). The full frequency range (0–0.25 Hz) of ALFF was divided into five different bands: slow-6 (0–0.01 Hz), slow-5 (0.01–0.027Hz), slow-4 (0.027–0.073 Hz), slow-3 (0.073–0.198 Hz), and slow-2 (0.198–0.25 Hz), (Buzsáki and Draguhn, 2004; Zuo et al., 2010). The signals of slow-6, slow-3, and slow-2 were discarded because they mainly reflect very low frequency drift, white matter (WM) signals, and high-frequency physiological noises, respectively, (Biswal et al., 1995; Zuo et al., 2010). Slow 4 and slow 5 were used for further analyses.

### Structural Data Analyses

To exclude the effect of structural damage on ALFF measurements, we performed a voxel-based morphometry (VBM) approach to compute the GM volume of each subject using DPARSF software. Briefly, cerebral tissues were segmented into GM, WM, and cerebrospinal fluid and were then normalized to the MNI space using a unified segmentation algorithm (Ashburner and Friston, 2005). T1 images were normalized to the MNI template using affine linear registration followed by Gaussian smoothing (FWHM = 6 mm). GM and WM volumes were calculated by estimating these segments. Brain parenchyma volume was calculated as the sum of GM and WM volumes.

### Statistical Analysis

Differences in demographic data between tinnitus patients and healthy controls were analyzed using between-group *t*-test for means and χ^2^-test for proportions (statistical significance was set at *p* < 0.05) using SPSS 18.0 software (SPSS, Inc., Chicago, IL, USA). To determine the effects of group, frequency band and interaction between frequency band and group on ALFF/fALFF, we performed a two-way repeated-measures analysis of variance (ANOVA) on a voxel-by-voxel basis with group (tinnitus patients and healthy controls) as a between-subject factor and frequency band (slow-4 and slow-5) as a repeated-measures variable using SPM8 software. All the statistical maps were corrected for multiple comparisons to a significance level of *p* < 0.05 by combining the individual voxel *p* < 0.05 with cluster size >3591 mm^3^ based on Monte Carlo simulations (Ledberg et al., 1998; Han et al., 2011).

Further correlative analyses between ALFF/fALFF values of significantly different brain regions and tinnitus characteristics were performed on the tinnitus groups by extracting the most significantly different frequency between groups using REST software. Then, Pearson’s correlation coefficients between abnormal ALFF/fALFF values and each clinical characteristic were analyzed using SPSS software. Partial correlations were calculated after correction for age, sex, and education. Given the large age range of the subjects in the current study, we performed a multiple regression in SPM8 software to investigate the potentially altered brain regions accompanied with increased age in each group.

## Results

### Structural Data

**Table 2** presents the comparisons of the whole-brain volumes (GM volume, WM volume, and brain parenchyma volume) between the tinnitus patients and the healthy controls. The results showed that there were no significant changes in GM and WM volumes between the two groups (*p* > 0.05).

**Table 2:** Comparisons of brain volumes in tinnitus patients and healthy controls.

	Tinnitus patients (*n* = 39)	Healthy controls (*n* = 41)	*p*-value
GM WM Brain parenchyma	585.82 ± 24.86 539.31 ± 27.39 1118.33 ± 32.26	576.93 ± 23.90 535.83 ± 30.50 1112.27 ± 40.36	0.107 0.594 0.461

*Data are presented as mean ± SD. GM, gray matter; WM, white matter.*

### ALFF Analyses

**FIGURE 1:**
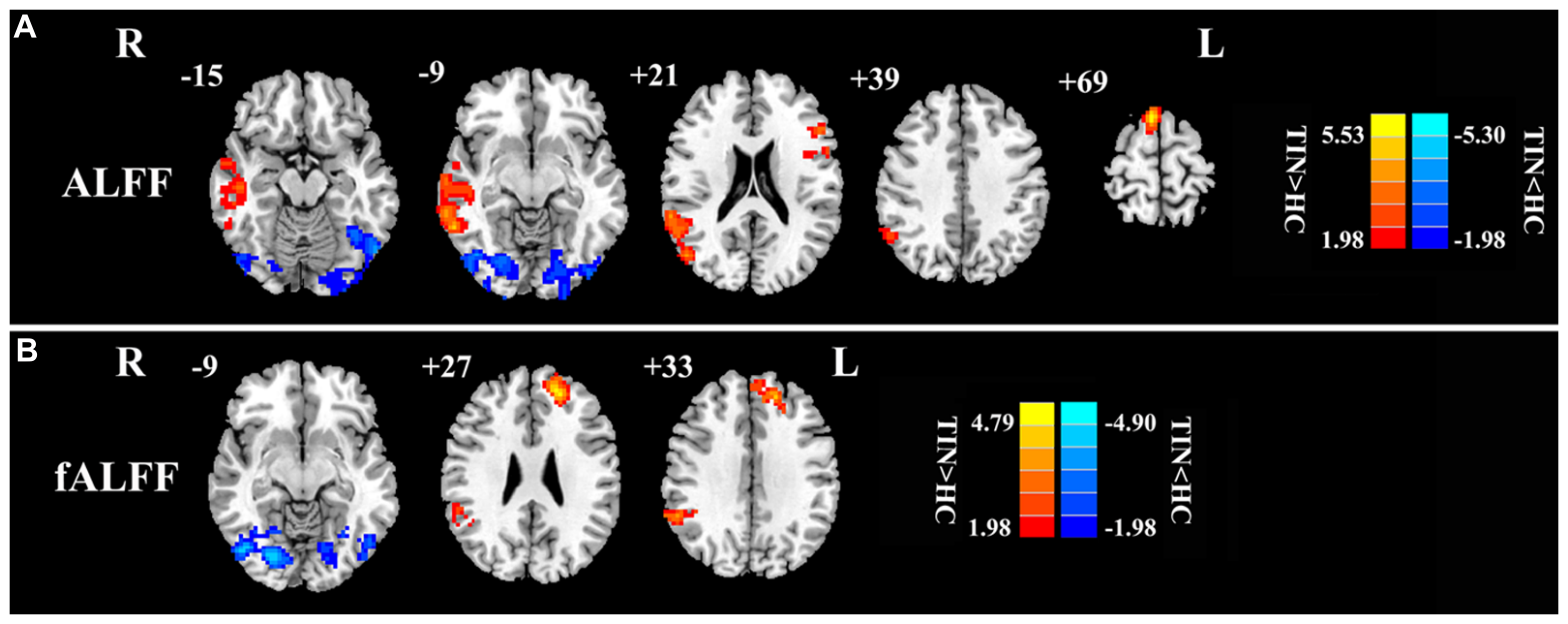
**The main effect of group on: **(A)** ALFF and **(B)** fALFF.** Red/hot colors represent higher ALFF (*t*-values 1.98 to 5.53; red to yellow, respectively) or fALFF (*t*-values 1.98 to 4.79; red to yellow, respectively) in the tinnitus group than in the control group while blue/cold colors represent lower ALFF (*t*-values –1.98 to –5.30; dark blue to light blue, respectively) or fALFF (*t*-values –1.98 to –4.90; dark blue to light blue, respectively) in the tinnitus group than controls. Results obtained by a two-way repeated-measure ANOVA. AlphaSim corrected *p* < 0.05 (individual voxel threshold *p* < 0.05 with a minimum cluster size of 3591 mm^3^). **Tables 3** and **4** show regions where significant increases and decreases occurred.

First, we examined the effect of tinnitus on ALFF values. Main effects from the two-way repeated-measure ANOVA are shown in **Figure 1**. Brain regions showing a significantly larger ALFF in tinnitus patients compared to healthy controls (TIN > HC, red colors) were seen in right SFG, right MTG, right AG, and left inferior frontal gyrus (IFG). Brain regions in which ALFF values were larger in healthy controls than in tinnitus patients (TIN < HC, blue colors) were found in bilateral middle occipital gyrus (MOG), (**Figure 1A** and **Table 3**).

**Table 3:** Result of group **×** frequency ANOVA of ALFF.

Brain region	BA	Peak MNI coordinates *x, y, z* (mm)	Peak *t*-value	Voxels
**Main effect of group**				
R middle temporal gyrus	21	60, –51, –9	4.9609	907
L inferior frontal gyrus	45	–51, 24, 21	4.0804	282
R angular gyrus	39	45, –75, 39	4.6951	143
R superior frontal gyrus	8	6, 15, 69	6.2468	783
B middle occipital gyrus	19	–54, –66, –15	–4.1240	472
**Group × frequency interaction**				
R orbitofrontal cortex	25	12, 18, –18	3.1698	375

*Corrected threshold of *p* < 0.05 determined by Monte Carlo simulation indicating a significant difference. BA, Brodmann’s area; MNI, Montreal Neurological Institute; L, left; R, right; B, bilateral.*

**FIGURE 2:**
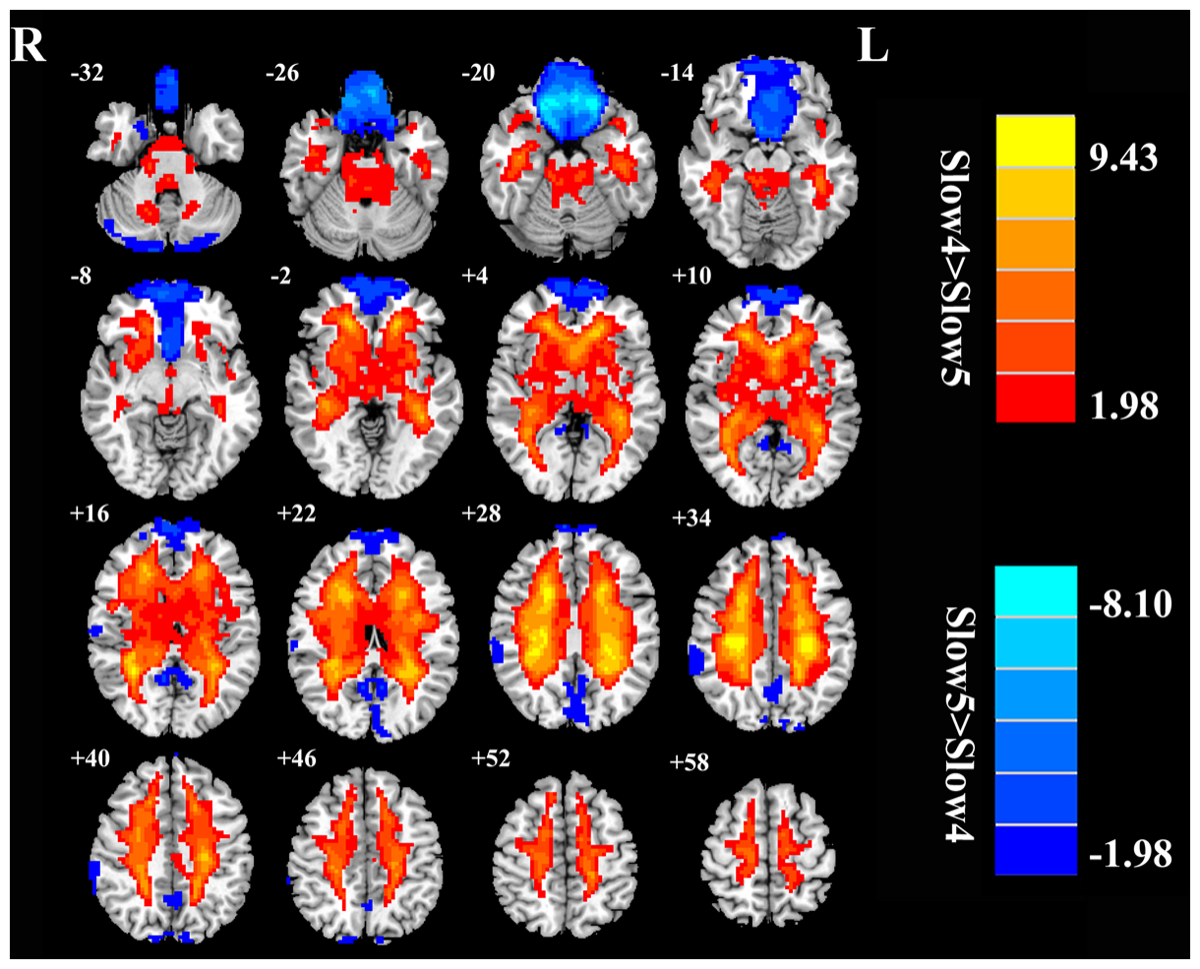
**The main effects of frequency band on ALFF.** Red/hot colors identify brain regions where slow-4 ALFF values were significantly greater than slow-5 ALFF values (*t*-values 1.98 to 9.43; red to yellow, respectively). Blue/cold colors identify brain regions where the slow-5 ALFF values were significantly greater than the slow-4 ALFF band values (*t*-values –1.98 to –8.10; dark blue to light blue, respectively). Results were obtained by a two-way repeated-measure ANOVA. AlphaSim corrected *p* < 0.05 (individual voxel threshold *p* < 0.05 with a minimum cluster size of 3591 mm^3^).

**FIGURE 3:**
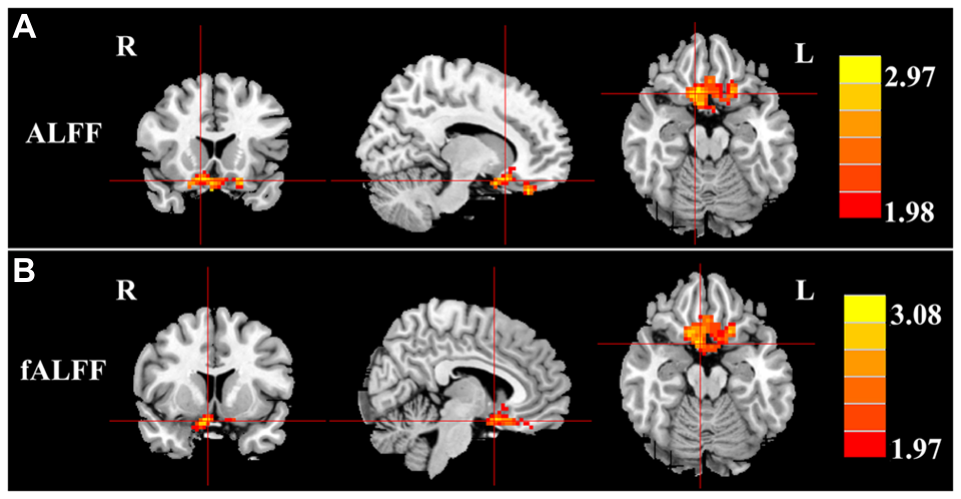
**Interaction between frequency bands and tinnitus vs. control groups on **(A)** ALFF values and **(B)** fALFF values in the orbitofrontal gyrus.** Red/hot colors identify brain regions where there was a significant interaction effect on ALFF (*t*-values 1.98 to 2.97; red to yellow, respectively) or fALFF (*t*-values 1.97 to 3.08; red to yellow, respectively). Results were obtained by a two-way repeated-measure ANOVA and *post hoc* test. AlphaSim corrected *p* < 0.05 (individual voxel thresholds *p* < 0.05 with a minimum cluster size of 3591 mm^3^).

**FIGURE 4:**
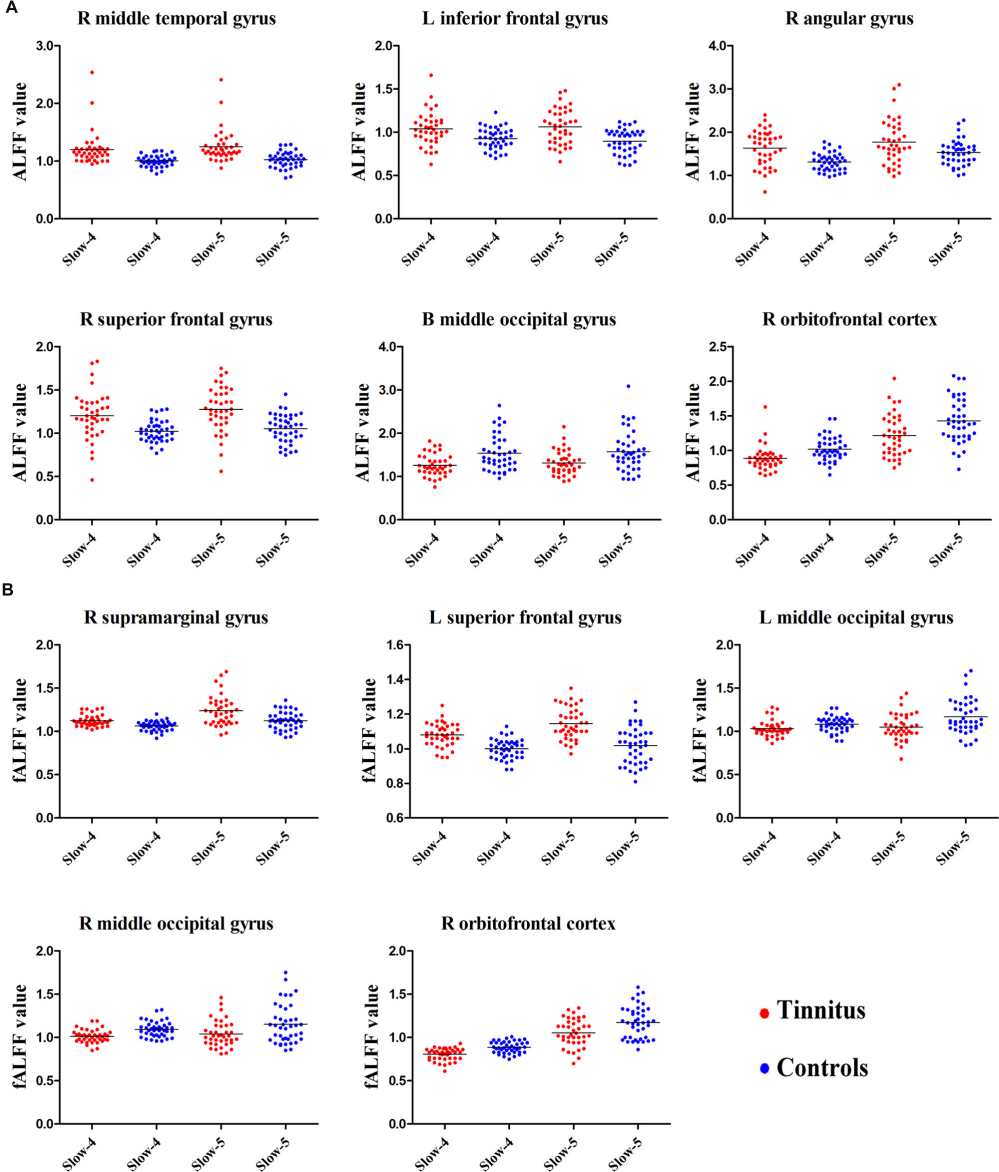
**The scatter plot showed the significant brain regions of abnormal **(A)** ALFF or **(B)** fALFF between tinnitus patients and healthy controls in slow-4 and slow-5 frequency bands.** The red dots represented tinnitus patients; the blue dots represented healthy controls. L, left; R, right; B, bilateral.

Next, we examined the data to determine the effect of slow-4 and slow-5 bands of ALFF (**Figure 2**). Brain regions showing a significantly larger slow-4 ALFF compared to slow-5 ALFF were found in the pons, midbrain, striatum, thalamus, hippocampus, and cerebellum (Slow 4 > Slow 5, red colors). In contrast, the values of slow-5 ALFF that were significantly larger than slow-4 ALFF occurred in the MFG, supramarginal gyrus (SMG), posterior cingulate cortex (PCC), and precuneus (Slow 5 > Slow 4, blue colors). In addition, we observed a significant interaction between frequency band and groups in the right orbitofrontal cortex (OFC; *p* < 0.05, AlphaSim corrected, **Figure 3A** and **Table 3**). **Figure 4A** showed the ALFF values of each significant brain regions between tinnitus patients and healthy controls in slow-4 and slow-5 frequency bands.

### fALFF Analyses

**FIGURE 5:**
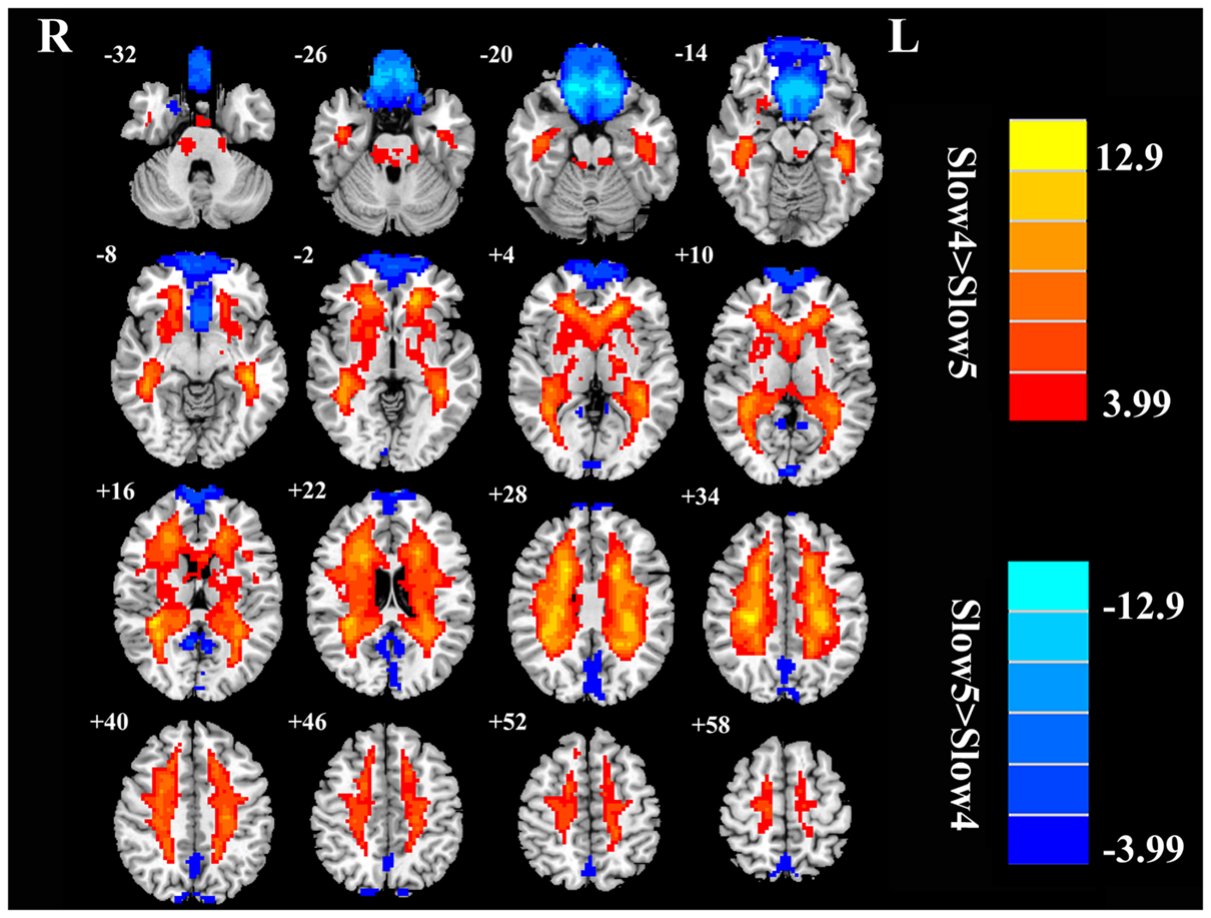
**The main effects for frequency band on fALFF.** Red/hot colors identify brain regions where slow-4 fALFF values were significantly greater than slow-5 fALFF values (*t*-values 3.99 to 12.9; red to yellow, respectively). Blue/cold colors identify brain regions where slow-5 fALFF values were significantly greater than slow-4 fALFF values (*t*-values –3.99 to –12.9; dark blue to light blue, respectively). Results were obtained by a two-way repeated-measure ANOVA. AlphaSim corrected *p* < 0.05 (individual voxel thresholds *p* < 0.05 with a minimum cluster size of 3591 mm^3^).

For the fALFF analyses that only included the slow-4 and slow-5 band, the brain regions showing a significant main effect/difference between tinnitus patients and controls (**Figure 1B** and **Table 4**) including the left SFG, right SMG (TIN > HC, red colors), and the bilateral MOG (TIN < HC, blue colors). The general locations of the main effects for slow-4 and slow-5 fALFF (**Figure 5**) were very similar to that in ALFF (**Figure 2**). In addition, we also observed significant interaction between frequency bands and groups in the right OFC (*p* < 0.05, AlphaSim corrected, **Figure 3B** and **Table 4**). **Figure 4B** showed the fALFF values of each significant brain regions between tinnitus patients and healthy controls in slow-4 and slow-5 frequency bands.

**Table 4:** Result of group × frequency ANOVA of fALFF.

Brain region	BA	Peak MNI coordinates *x, y, z* (mm)	Peak *t*-value	Voxels
**Main effect of group**				
R supramarginal gyrus	40	57, –48, 33	3.6976	260
L superior frontal gyrus	10	–21, 45, 27	5.3486	781
L middle occipital gyrus	19	–51, –69, –9	–3.3196	269
R middle occipital gyrus	19	42, –72, –9	–4.8310	437
**Group × frequency interaction**				
R orbitofrontal cortex	25	6, 9, –18	3.3001	314

*Corrected threshold of *p* < 0.05 determined by Monte Carlo simulation indicating a significant difference. BA, Brodmann’s area; MNI, Montreal Neurological Institute; L, left; R, right.*

### Correlation Analysis Results

**FIGURE 6:**
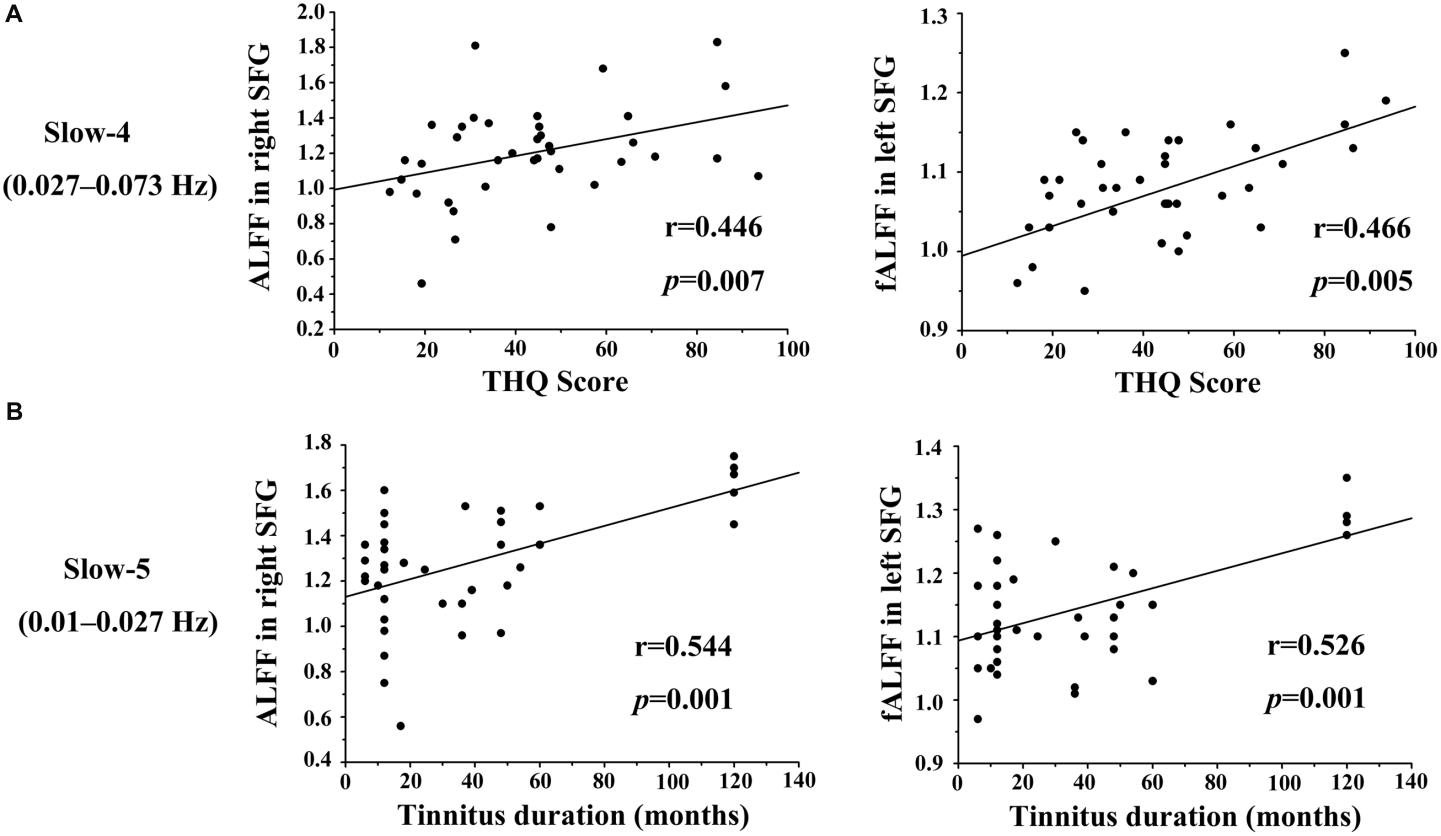
**Significant correlations observed between the ALFF/fALFF in two frequency bands vs. THQ score and tinnitus duration. (A)** Slow-4 ALFF values in right SFG (left) and fALFF values in left SFG (right) were positively correlated with THQ scores (*r* = 0.446, *p* = 0.007; *r* = 0.466, *p* = 0.005). **(B)** Slow-5 ALFF values in right SFG (left) and fALFF values in left SFG (right) were positively correlated with tinnitus duration (*r* = 0.544, *p* = 0.001; *r* = 0.526, *p* = 0.001).

We calculated the correlations between aberrant slow-4 and slow-5 bands in ALFF/fALFF and tinnitus characteristics to determine if there were any significant relationships. In tinnitus patients, slow-4 ALFF in right SFG (**Figure 6A**, left panel, *r* = 0.446, *p* = 0.007) and slow-4 fALFF in left SFG (**Figure 6A**, right panel, *r* = 0.466, *p* < 0.005) were positively correlated with THQ scores. Moreover, slow-5 ALFF in right SFG (**Figure 6B**, left panel, *r* = 0.544, *p* = 0.001) and slow-5 fALFF in left SFG (**Figure 6B**, right panel, *r* = 0.526, *p* = 0.001) were positively correlated with tinnitus duration. The ALFF activity in right SFG was not correlated with the fALFF in left SFG (Supplementary Figure S1). The other significant increases or decreases in ALFF or fALFF values were not significantly related to THQ scores or tinnitus duration (**Table 5**). We also did not observe any significant brain regions affected by increase of age in each group. In addition, the tinnitus severity was not significantly correlated with tinnitus duration through a multiple regression analysis (Supplementary Figure S2).

**Table 5:** Correlation coefficients between aberrant ALFF/fALFF in slow-4/slow-5 bands and tinnitus characteristics.

Brain region	Tinnitus duration	THQ score
**(I) ALFF**	**Slow-4/Slow-5**	**Slow-4/Slow-5**
R superior frontal gyrus	0.178/**0.544**^∗^	**0.446**^∗^/0.145
R middle temporal gyrus	–0.090/–0.061	0.030/0.126
L inferior frontal gyrus	–0.080/–0.175	0.018/–0.017
R angular gyrus	–0.039/–0.014	–0.019/0.043
B middle occipital gyrus	–0.009/0.117	–0.070/–0.094
**(II) fALFF**		
L superior frontal gyrus	0.102/**0.526**^∗^	**0.466**^∗^/0.193
R supramarginal gyrus	–0.186/–0.129	–0.149/0.190
L middle occipital gyrus	0.065/0.118	0.049/–0.124
R middle occipital gyrus	0.147/0.214	–0.026/0.020

*Correlations were controlled for age, sex, and education. ^∗^*P* < 0.05. L, left; R, right; B, bilateral. Bolded values represent the significant correlation coefficients.*

## Discussions

To our knowledge, this is the first study to examine changes of LFO amplitudes (ALFF and fALFF) in chronic tinnitus at two different frequency bands (slow-4 and slow-5 bands). We found significant differences between tinnitus patients and matched controls in ALFF/fALFF slow-4 and slow-5 bands in several brain regions. The ALFF and fALFF in the slow-4 band were higher in the midbrain, basal ganglia, hippocampus and cerebellum, and lower in the MFG, SMG, PCC, and precuneus, in comparison to that in slow-5. In addition, the right OFC exhibited significant interaction in ALFF/fALFF between frequency bands and groups. Importantly, aberrant ALFF/fALFF activity in the SFG at slow-4 and slow-5 bands was correlated with tinnitus duration and tinnitus distress. Our results suggest that the abnormal spontaneous LFO amplitudes in chronic tinnitus patients are frequency dependent.

In the current study, two different kinds of metrics (ALFF and fALFF) were used to investigate the changes of LFO amplitudes in tinnitus patients in slow-4 and slow-5 bands. Surprisingly, the abnormalities of both ALFF and fALFF activity were similar in many brain regions, especially in the SFG, which was significantly correlated with the tinnitus characteristics. ALFF is calculated as the sum of amplitudes within a specific low frequency range (0.01–0.1 Hz) while fALFF is calculated as a fraction of the sum of amplitudes across the entire frequency range (0–0.25 Hz) detectable in a given signal (Zang et al., 2007; Zou et al., 2008). These two are promising quantitative methods for measuring the LFO amplitudes and detecting spontaneous brain activity. Although both ALFF and fALFF are sensitive mostly to signal from GM region, ALFF is more prone to noise from physiological sources, particularly near the ventricles and large blood vessels, and these are attenuated in fALFF (Zou et al., 2008; Zuo et al., 2010). Anyway, both algorithms have been applied to evaluate the LFO amplitudes of normal and pathological brains (Yang et al., 2007; Zang et al., 2007; Han et al., 2011; Yu et al., 2014). However, the increased specificity to the GM signal for fALFF compared to ALFF may suggest favoring the former, but will reduce test–retest reliability, making fALFF intrinsically less reliable (Arndt et al., 1991; Zuo et al., 2010). Thus, in order to maximize the reliability across subjects while providing sufficient specificity to examine individual differences, it is probably most advisable to report findings with both measures (Zuo et al., 2010). Further studies will be required to determine which method is more effective and better for identifying aberrant spontaneous LFO amplitudes in tinnitus remains an important question for future work.

Our group effect analyses showed that the ALFF was higher in right SFG, right MTG, left IFG, and right AG while the fALFF was higher in left SFG and right SMG, but both were lower in bilateral MOG in tinnitus patients than in healthy controls (**Figure 1**; **Tables 3** and **4**). These results are consistent with our earlier report using ALFF in the typical frequency band (0.01–0.08 Hz; Chen et al., 2014). Using single photon emission computed tomography (SPECT) and positron emission tomography (PET), previous studies have demonstrated that tinnitus patients exhibited hypermetabolism and hyperperfusion in the MTG (Mirz et al., 1999; Plewnia et al., 2007b; Farhadi et al., 2010), AG (Mirz et al., 1999; Plewnia et al., 2007a; Song et al., 2012), and SMG (Mirz et al., 1999; Plewnia et al., 2007a) confirming the involvement of these structures for tinnitus. More importantly and in a broader context, these brain regions are components of the DMN. The DMN, which consists of nodes in the MTG, MFG, PCC, precuneus, and inferior parietal lobe, is most active at rest and shows reduced activity when a subject enters a task-based state involving attention or goal-directed behavior (Raichle et al., 2001; Mantini et al., 2007). Using resting-state fMRI, several studies have found disrupted spontaneous neuronal activity and functional connectivity in the DMN regions in tinnitus patients (Burton et al., 2012; Maudoux et al., 2012b; Schmidt et al., 2013; Chen et al., 2014, 2015a,b; Zhang et al., 2015). Thus, we speculate that the phantom sound of tinnitus, particular severe cases that cause anxiety or distress, may disrupt the resting-state DMN. However, the source or type of aberrant spontaneous neuronal activity within DMN regions due to tinnitus remains unknown.

Several studies have reported that the frontal cortex plays a pivotal role in tinnitus (Jastreboff, 1990; Lanting et al., 2009; Vanneste et al., 2012). Consistent with this view we found increased ALFF/fALFF in the SFG. Besides the significant group difference, significant positive correlations were observed between increased ALFF/fALFF in bilateral SFG and tinnitus duration or tinnitus distress in both slow-4 and slow-5 bands (**Figure 6**). Other neuroimaging studies have identified abnormalities in the frontal cortex associated with tinnitus (Lanting et al., 2009; Schecklmann et al., 2013; Chen et al., 2014). Rauschecker et al. (2010) found structural and functional differences between tinnitus patients and controls in ventromedial prefrontal cortex; these differences were related to the subjective loudness of tinnitus (Leaver et al., 2011). Using PET, a significant increase in metabolism was seen in the SFG of tinnitus (Mirz et al., 2000a), some of whom could modulate the loudness of their tinnitus (Lockwood et al., 2001). In several task-based fMRI studies, the SFG was found to be activated in healthy subjects by aversive acoustic stimuli, suggesting that the negative affect associated with tinnitus may involve this area of the cortex (Mirz et al., 2000b). These results are consistent with greater activation in the STG of tinnitus patients during a pitch-discrimination task in an emotional context (Wunderlich et al., 2009). While it is difficult to establish conclusive interpretations from the correlation results, we speculate that the integration of multi-sensory information in tinnitus perception may be influenced by enhanced LFO amplitudes in the SFG.

We also observed reduced ALFF/fALFF activity in bilateral MOG in both frequency bands, which is compatible with previous fMRI studies showing aberrant function in visual network in tinnitus patients (Burton et al., 2012; Maudoux et al., 2012b; Chen et al., 2014, 2015a,b). The multisensory connections between auditory and visual regions make it possible for external acoustic stimuli or the internal phantom sound of tinnitus to alter the brain activity in the visual areas (Wang et al., 2008; Cate et al., 2009; Kayser et al., 2009). Thus, decreased LFO amplitudes may be due to increased attention devoted to processing a phantom sound which decreases neural synchrony or interaction with the visual system (Meredith et al., 2012; Araneda et al., 2015). These results suggest that further work is needed to determine how the phantom sound of tinnitus disrupts the normal audio–visual interactions.

Our frequency selective analyses showed that both ALFF and fALFF were higher in the slow-4 band than in the slow-5 band mainly in subcortical regions that included the pons, midbrain, striatum, thalamus, and hippocampus, but were lower in several DMN regions including the MFG, SMG, PCC, and precuneus (**Figures 2** and **5**). Although the origins, relation, and specific physiological functions of slow-4 and slow-5 frequency bands have not been fully clarified, neighboring frequency bands within the same neuronal network may compete or interact with each other (Engel et al., 2001). Previous studies suggest that LFO amplitudes arise from spontaneous neural activity which generated their rhythmic patterns through information exchange with neighboring brain regions to facilitate specific response characteristics (Biswal et al., 1995). The period of an oscillation is constrained by the size of neuronal pool engaged in a given cycle since most neuronal connections are local (Csicsvari et al., 2003; Buzsáki and Draguhn, 2004). Increasing evidences indicate that slow-4 band ALFF is greater than the slow-5 band in subcortical regions (Zuo et al., 2010; Han et al., 2011; Hou et al., 2014; Yu et al., 2014). However, slow-5 band ALFF has higher amplitude than slow-4 band mainly in the DMN (Zuo et al., 2010; Han et al., 2011; Wei et al., 2014). The lower frequency band has the highest power, and localizes mainly to prefrontal, parietal, and occipital cortex; the higher frequency band has less power and localizes mainly in subcortical structures (Baria et al., 2011). Buzsáki and Draguhn (2004) noted that the cortical regions have relatively large-amplitude low-frequency neural oscillations and that contribute to long distance connections in large networks such as the DMN. Subcortical regions have higher frequency, fast local events that are modulated by widespread slow oscillations. Csicsvari et al. (2003) suggested that cortical regions exert strong descending influences over subcortical regions in keeping with the large number of descending projections from auditory cortical regions to brainstem (Mellott et al., 2014). Taken together, these results suggest that it may be informative to investigate tinnitus-related neural alterations in specific frequency bands in cortical areas to determine if they influence or are correlated with frequency specific changes in the brainstem, i.e., cortical modulation of brainstem activity. The slow-4 band is more sensitive to detect abnormalities of intrinsic brain activity in the subcortical regions while the slow-5 band is more sensitive for the DMN regions in tinnitus patients compared to the other bands. Such frequency specific fluctuations will help understand the neuropathological basis underlying tinnitus and monitor disease progression in future studies.

The role of the OFC in tinnitus remains poorly understood. However, previous neuroimaging studies have identified alterations in the OFC of tinnitus patients in terms of the functional coupling of long-range cortical networks (Schlee et al., 2009; Vanneste et al., 2012; Song et al., 2014). The OFC is considered part of the reward system (Tremblay and Schultz, 1999; Rolls, 2004; Kringelbach, 2005) and in this capacity it may contribute to the aversive aspects of tinnitus. Interestingly, according to our interaction results (**Figure 3**; **Tables 3** and **4**), LFO amplitudes in right OFC were modulated not only by disease but also by frequency band. Future study with combination of fMRI and electrophysiological methods would be helpful for understanding the underlying neural mechanisms of the interaction in temporal and frequency domains.

Tinnitus is often perceived as tonal or narrow band noise suggesting that the neural generator for tinnitus resides within a discrete tonotopic region in the auditory pathway. However, we observed functional changes in non-tonotopic beyond the auditory pathway such as SFG, which is involved in self-awareness (Goldberg et al., 2006), and OFC. How could these non-auditory areas contribute to tonal tinnitus? The prefrontal cortex and the OFC are associated with auditory attention (Blood et al., 1999; Voisin et al., 2006) and appear to exert broad inhibitory control over primary auditory cortex and other central auditory structures (Knight et al., 1989; Angrilli et al., 2008); this allows for a broad, generalized top-down modulation of auditory processing allowing the system to focus on different features of the acoustic scene or internal states (Norena et al., 1999; Goldberg et al., 2006). Dysregulation of an expansive, top-down inhibitory mechanisms could “open the gate” (Rauschecker et al., 2010), allowing narrowband or wideband aberrant tinnitus signals within the auditory pathway to become more salient and enter consciousness (Knight et al., 1989; Goldberg et al., 2006).

We failed to observe significant differences in whole brain GM volume as well as regional GM volume in any of the regions showing altered ALFF/fALFF in tinnitus patients. In contrast, previous studies have reported increases and decreases in GM volume in auditory and non-auditory regions in tinnitus patients (Adjamian et al., 2014). However, the changes in GM volume seen in these tinnitus patients were typically correlated with hearing loss particularly when testing was extended beyond 8 kHz. Since our subjects had normal hearing out to 16 kHz and no evidence of hyperacusis; thus likely accounts for the lack of change in GM volume in our tinnitus subjects. An alternative possibility is that our analytical methods are not sensitive enough to detect the structural differences between neuroanatomical structures in our tinnitus patients versus controls. In any case, our results suggest that frequency-specific alterations in ALFF/fALFF can occur prior to any obvious structural changes in tinnitus patients with normal hearing and absence of hyperacusis.

Close inspection of **Figure 6B** reveals five subjects in which tinnitus duration exceeded 100 months and these five had ALFF and fALFF values that were equal to or slightly larger than the range of values of subjects that shorter tinnitus durations. This raises the possibility that these five subjects were so-called outliers and largely responsible for the significant correlation in **Figure 6B**. While we have not *a priori* reason for excluding the data from these five subjects, or any other subjects at the other extreme end of the distribution (e.g., small ALFF or fALFF values and short tinnitus duration), we nevertheless recomputed the correlations between tinnitus duration and ALFF or fALFF in the SFG, and found positive but not significant correlations. Since the correlations were no longer significant, one interpretation of these results is that tinnitus duration must exceed some critical duration (>60 months) in order for ALFF or fALFF to increase significantly in the SFG, i.e., there may be a critical duration for such effects to occur.

Several factors limit the generalizability and interpretation of our results. First, due to the relatively small sample size, we did not sort the fMRI data into left, right or bilateral-sided tinnitus to see whether and how the laterality of tinnitus contributed to the lateralization of neural effects. Therefore, additional studies are required with a larger sample size of different lateralization of the tinnitus percept. Second, although we have attempted to minimize the amount of scanner noise with earplugs, the subjects cannot be completely prevented from hearing sounds generated by the scanner would activate the auditory pathway thereby disrupting the pattern of neural activity that would occur in a quiet environment (Logothetis et al., 2009). The acoustic characteristics of the noise generated by the MRI scanner may influence the magnitude and location of slow-4 and slow-5 ALFF/fALFF activity. Moreover, tinnitus patients could have been experiencing their tinnitus perception during the scan, which could affect the fMRI results. We did not ask the subjects if they experienced tinnitus during the scan because instructing tinnitus patients to listen to their tinnitus would have activated the attentional networks. A post-scan questionnaire will be included to determine whether the tinnitus perception is experienced in the scanner in order to identify its potential effect on brain activity in our future experiments. Finally, our study only investigated between-group ALFF/fALFF changes in the slow-4 and slow-5 frequency bands. Further investigations exploring the ALFF/fALFF changes in all frequency sub-bands and their relationships with clinical tinnitus characteristics could reveal additional insights on the neural basis of tinnitus (Baliki et al., 2011; Baria et al., 2011).

## Conclusion

In this study, chronic tinnitus patients with normal hearing exhibited abnormal LFO amplitudes in several brain regions including the DMN regions, prefrontal cortex and visual cortex. Enhanced ALFF/fALFF values in bilateral SFG were positively correlated with tinnitus duration and tinnitus distress at both slow-4 and slow-5 bands. Furthermore, the significant interaction in the OFC indicated that LFO amplitudes abnormalities in chronic tinnitus were frequency-dependent. Our results suggest that frequency-specific analysis of ALFF/fALFF could provide new and novel insights related to various auditory and non-auditory dimensions of tinnitus.

## Conflict of Interest Statement

The authors declare that the research was conducted in the absence of any commercial or financial relationships that could be construed as a potential conflict of interest.

## Footnotes

1 http://www.fil.ion.ucl.ac.uk/spm

2 http://www.restfmri.net
