# Supplementary material for: Frequency-specific alternations in the amplitude of low-frequency fluctuations in chronic tinnitus
Source: Front Neural Circuits. 2015 Oct 29;9:67. doi: 10.3389/fncir.2015.00067 (PMC4624866; doi:10.3389/fncir.2015.00067)
Supplement: Supplementary file 1 [file Data_Sheet_1.DOCX]

**Supplementary Materials**


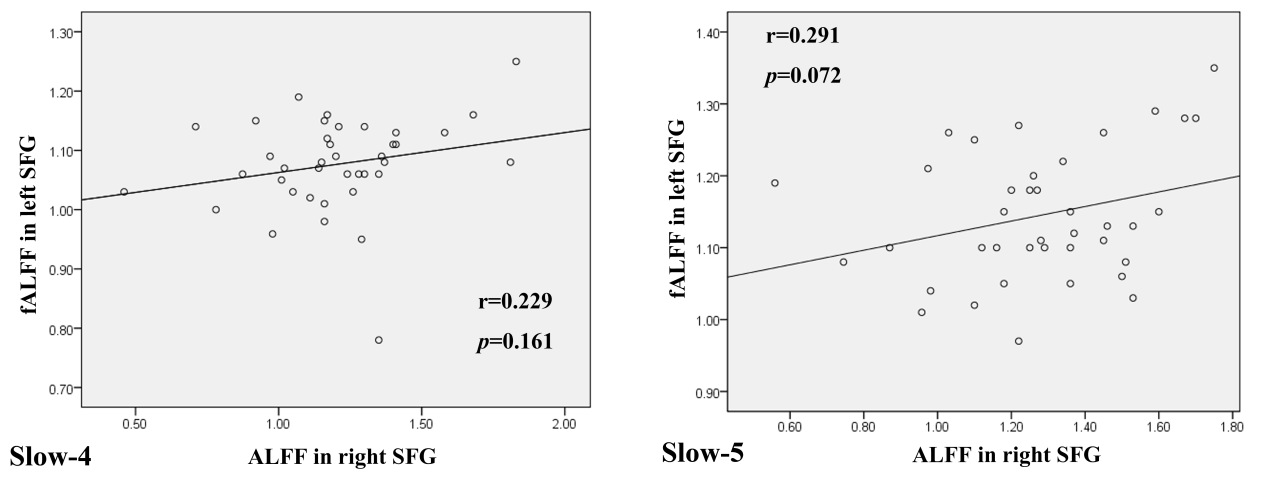


**Supplementary** **Figure 1:** No significant correlations were detected between the ALFF in right SFG and fALFF in left SFG at both slow-4 and slow-5 bands (*p*>0.05).


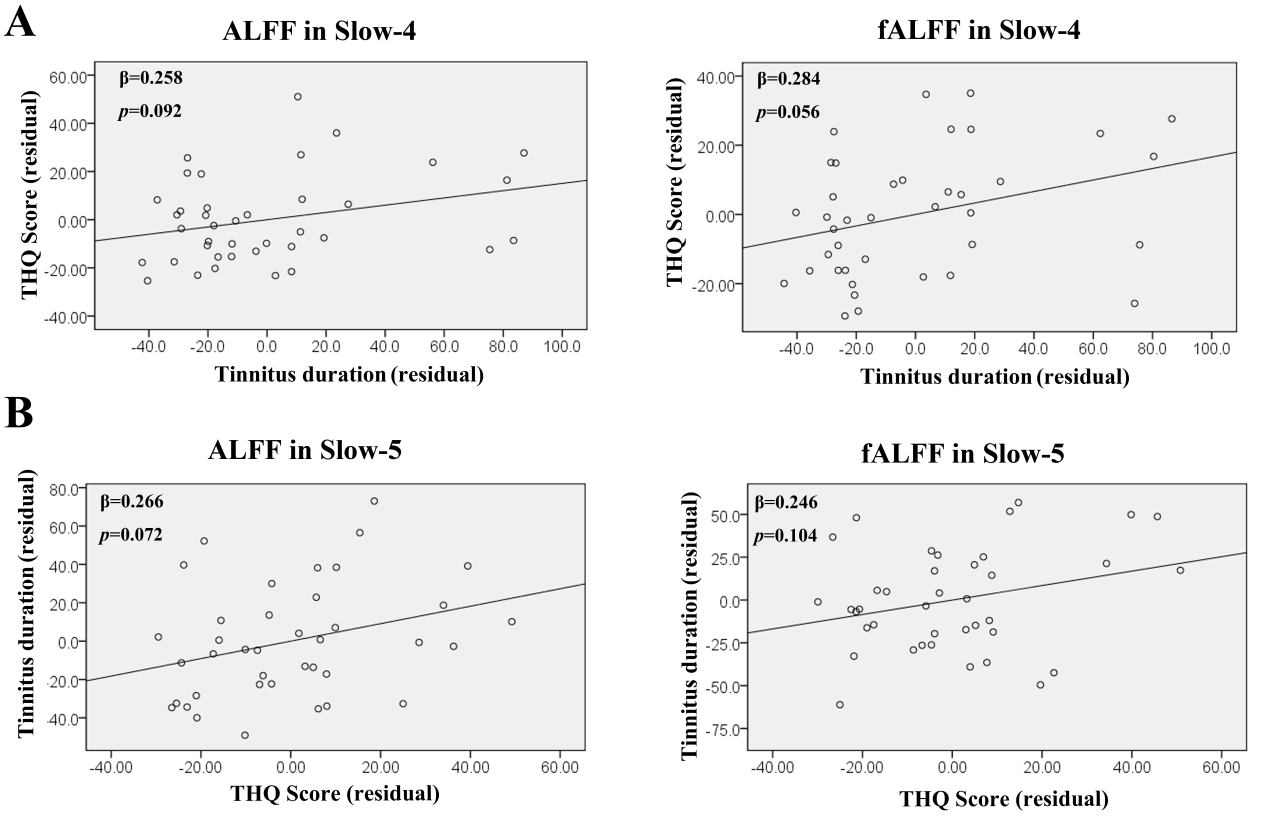


**Supplementary** **Figure 2:** A multiple regression analysis accounting for THQ score and tinnitus duration. (A) The THQ score was not significantly correlated with tinnitus duration in ALFF and fALFF at slow-4 band (*p*>0.05). (B) The tinnitus duration was not significantly correlated with THQ score in ALFF and fALFF at slow-5 band (*p*>0.05).
